# Flavanols and Platelet Reactivity

**DOI:** 10.1080/10446670410001722140

**Published:** 2005-03

**Authors:** Debra A. Pearson, Roberta R. Holt, Dietrich Rein, Teresa Paglieroni, Harold H. Schmitz, Carl L. Keen

**Affiliations:** 1 Department of Human Biology ES 301 University of Wisconsin-Green Bay 2420 Nicolet Drive Green Bay WI 54311 USA; 2 Department of Nutrition University of California One Shields Avenue Davis CA 95616 USA; 3 Sacramento Medical Foundation Center for Blood Research 1625 Stockton Boulevard Sacramento CA 95816 USA; 4 Mars Incorporated 800 High Street Hackettstown NJ 07840 USA; 5 Department of Internal Medicine University of California One Shields Avenue Davis CA USA

## Abstract

Platelet activity and platelet-endothelial cell interactions are important in the acute development of thrombosis, as well as in the pathogenesis of cardiovascular disease. An increasing number of foods have been reported to have platelet-inhibitory actions, and research with a number of flavanol-rich foods, including, grape juice, cocoa and chocolate, suggests that these foods may provide some protection against thrombosis. In the present report, we review a series of *in vivo* studies on the effects of flavanol-rich cocoa and chocolate on platelet activation and platelet-dependent primary hemostasis. Consumption of flavanol-rich cocoa inhibited several measures of platelet activity including, epinephrine- and ADP-induced glycoprotein (GP) IIb/IIIa and P-Selectin expression, platelet microparticle formation, and epinephrine-collagen and ADP-collagen induced primary hemostasis. The epinephrine-induced inhibitory effects on GP IIb/IIIa and primary hemostasis were similar to, though less robust than those associated with the use of low dose (81 mg) aspirin. These data, coupled with information from other studies, support the concept that flavanols present in cocoa and chocolate can modulate platelet function through a multitude of pathways.


*Clinical & Developmental Immunology*, March 2005; 12(1): 1–9

Taylor & Francis

Taylor & Francis Group

### Flavanols and platelet reactivity

DEBRA A. PEARSON[1], ROBERTA R. HOLT[2], DIETRICH REIN[2], TERESA PAGLIERONI[3], HAROLD H. SCHMITZ[4], & CARL L. KEEN[2,5]

[1]*Department of Human Biology ES 301, University of Wisconsin-Green Bay, 2420 Nicolet Drive, Green Bay, WI 54311, USA,* [2]*Department of Nutrition, University of California, One Shields Avenue, Davis, CA 95616, USA,* [3]*Sacramento Medical Foundation, Center for Blood Research, 1625 Stockton Boulevard, Sacramento, CA 95816, USA,* [4]*Mars Incorporated, 800 High Street, Hackettstown, NJ 07840, USA, and* [5]*Department of Internal Medicine, University of California, One Shields Avenue, Davis, CA, USA*

**Abstract**

Platelet activity and platelet-endothelial cell interactions are important in the acute development of thrombosis, as well as in the pathogenesis of cardiovascular disease. An increasing number of foods have been reported to have platelet-inhibitory actions, and research with a number of flavanol-rich foods, including, grape juice, cocoa and chocolate, suggests that these foods may provide some protection against thrombosis. In the present report, we review a series of *in vivo* studies on the effects of flavanol-rich cocoa and chocolate on platelet activation and platelet-dependent primary hemostasis. Consumption of flavanol-rich cocoa inhibited several measures of platelet activity including, epinephrine- and ADP-induced glycoprotein (GP) IIb/IIIa and P-Selectin expression, platelet microparticle formation, and epinephrine-collagen and ADP-collagen induced primary hemostasis. The epinephrine-induced inhibitory effects on GP IIb/IIIa and primary hemostasis were similar to, though less robust than those associated with the use of low dose (81 mg) aspirin. These data, coupled with information from other studies, support the concept that flavanols present in cocoa and chocolate can modulate platelet function through a multitude of pathways.

**Keywords:** *Cocoa flavanols, flavonoids, chocolate, platelet activation, cardiovascular disease*

### Introduction

Platelet activity and platelet-endothelial cell interactions are important not only in the acute development of thrombosis, but also in the pathogenesis of cardiovascular disease (CVD) (Osterud 1997, Rauch et al. 2001, Ruggeri, 2002). Frequently, the first clinical manifestation, and often-fatal event, of CVD is thrombi release from unstable atherosclerotic plaque, resulting in myocardial ischemia/infarction or stroke. Thus, antiplatelet therapies such as, aspirin (ASA), clopidogrel and glycoprotein IIb/IIIa inhibitors, are important components of therapeutic regimes for conditions associated with vascular pathology (Schafer 1996, Hennekens 1997, Bhatt and Topol 2000, Rauch et al. 2001). However, most of these therapies are employed to prevent secondary, rather than primary thrombotic events. Interestingly, an increasing number of foods and beverages (e.g. fish rich in omega-3 fatty acids, onions, tea, grape juice and garlic) have been observed to have platelet-inhibitory actions (Srivastava 1986, Apitz-Castro et al. 1992, Schafer 1996); and research with a number of flavanol-rich foods and beverages, such as grape juice, tea, cocoa and chocolate, suggests that these foods be particularly beneficial with respect to platelet function (Demrow et al. 1995, Pace-Asciak et al. 1996, Osman et al. 1998, Keevil et al. 2000, Freedman et al. 2001, Kris-Etherton and Keen 2002).

Historically, the formulation of dietary guidelines has been based, in part, by describing diets that will provide adequate amounts of vitamins, minerals, essential fatty acids and fiber that will maintain health. In programs of chronic disease prevention, such as CVD, diet is considered an integral component,

Correspondence: C.L.Keen, Department of Nutrition, University of California, One Shields Avenue, Davis, CA 95616, USA. Tel: 1 530 752 6331. Fax: 1 530 752 8966. E-mail: clkeen@ucdavis.edu

ISSN 1740-2522 print/ISSN 1740-2530 online © 2005 Taylor & Francis Group Ltd.
DOI: 10.1080/10446670410001722140

especially with regards to weight loss, in order to achieve desirable levels of blood lipids, and pressure (Krauss et al. 2000, Hu and Willett 2002). In addition to the maintenance of a healthy body weight, current American Heart Association recommendations include the daily consumption of a variety of fruits and vegetables and grain products (Krauss et al. 2000). Such foods contain vitamins, minerals and macronutrients that are known to provide certain health benefits, however, there is an increasing recognition of the fact that some foods, including fruits and vegetables, provide a greater, or different, health benefits than others. In an effort to better understand the different health properties of foods increasing attention has been given to the characterization of a wide number of plant polyphenolic compounds, including those in the flavonoid class. It is now recognized that flavonoids can affect a variety of enzymatic, and inter- and intracellular systems which can modulate immune function, inflammatory processes, vascular reactivity, antioxidant mechanisms, cell proliferation and platelet function (Middleton et al. 2000), thus, making these nutrients important components of the nutritional profile of the diet.

The flavonoids are the largest and most widely distributed class of phytochemicals, and can be found in foods such as red wine, tea, grapes, cocoa and chocolate (Kris-Etherton and Keen 2002). Certain cocoas and chocolate are unusual in that they can contain very high amounts of the flavanol monomers epicatechin and catechin, and their oligomers known as procyanidins (Hammerstone et al. 1999). In this paper, we review some of the effects of flavanols found in cocoa and chocolate on platelet function, and their potential as cardioprotective dietary agents.

### *Potential cocoa components, exclusive of flavonoids, that influence platelet reactivity*

Prior to an in depth discussion of the potential effects that cocoa flavonoids can have of platelet function, it is important to note that several other components in chocolate can potentially affect platelet function and cardiovascular health. In addition to the flavonoids, foods derived from cocoa are rich in minerals, lipid, and other non-flavonoid-based phytochemicals (Chevaux et al. 2001, Rios et al. 2003, Steinberg et al. 2003). Several minerals that are found in large amounts in cocoa may potentially provide some protection against platelet activation and thrombus formation. When measured on a per serving basis, dark and milk chocolate (44 g/serving) contain an equal or greater amounts of potassium, magnesium and calcium than do apples, red wine and cranberry juice (Steinberg et al. 2003). High potassium intakes have been associated with a reduced risk for CVD (Young et al. 1995), currently 3500 mg/day is recommended for primary prevention of hypertension (Whelton et al. 2002). In addition, potassium has been reported to decrease lesion formation after balloon angioplasty in rat and swine models, and reduce thrombosis formation in dogs as measured by the Folts model of coronary artery thrombosis (Lin and Young 1994, Ma et al. 2000, 2001). The magnesium concentration in chocolate and cocoa can be substantial being sufficient to correct chronic magnesium deficiencies in certain animal models (Planells et al. 1997). With regards to platelet function, Hughes and Tonks (Hughes and Tonks 1966) first observed that magnesium reduced ADP-induced platelet aggregation. In addition, magnesium has been reported to decrease collagen-induced platelet activation, possibly through changes in membrane fluidity and reduced thromboxane formation (Sheu et al. 2002). Platelet activation and aggregation is accompanied with increases in intracellular calcium, leading to activation of several different platelet secondary messenger and enzyme systems. Thus, a high dietary intake of calcium could potentially affect numerous platelet systems. Attenuation in platelet aggregation was observed in spontaneously hypertensive rats after calcium supplementation (Otsuka et al. 1997), while in human subjects calcium supplementation decreased intraplatelet free calcium (Petrov and Lijnen, 1999).

Due to the high cocoa butter content of cocoa and chocolate, these foods are sometimes viewed as “bad foods” with regard to vascular health. However, while 60% of the fat in cocoa butter exists as saturated stearic and palmitic acid (Otton et al. 1998, Steinberg et al. 2003), studies to date have typically shown neutral, or positive effects of cocoa consumption on plasma lipid markers (Kris-Etherton and Mustad 1994, Saamman et al. 2000, Wan et al. 2001). A recent meta-analysis of 60 studies concluded that palmitic acid had little effect on the ratio of total to HDL cholesterol, while stearic acid tended to reduce this ratio (Mensink et al. 2003). However, mixed results have been reported when studies examined the effects of individual fatty acids on platelet function, with particular regards to stearic acid. Initial studies reported a thrombogenic effect of stearic acid when it was injected directly into animal models; although this effect was muted when the stearic acid was mixed with albumin (Hook 1994). A correlation was found between stearic acid intake and coagulation protein factor VII in subjects given diets high in saturated or unsaturated fat, or a high carbohydrate diet (Mitropoulos et al. 1994). However, factor VII activation was decreased in subjects given diets rich in stearic acid compared to subjects given diets rich in palmitic acid (Tholstrup et al. 1994). The eicosanoids, prostacyclin and thromboxane, are potent platelet modulators formed by cyclooxygenase from arachidonic acid. While prostacyclin helps maintain the platelets in an acquiescent state, thromboxane potentiates platelet activation. Although a decrease in arachidonic acid concentration was observed in platelet phospholipids after subjects consumed cocoa butter or chocolate as a stearic acid source (Mustad et al. 1993), urinary thromboxane and prostacyclin levels remain unchanged following the consumption of diets rich in stearic acid (Mustad et al. 1993, Blair et al. 1994).

### Flavonoids

Cocoa and chocolate can be particularly rich in flavonoids, specifically the flavonoid subclass flavan-3-ol (flavanol) and oligomers of the flavanols epicatechin and catechin known as the proanthocyanidins (procyanidins). Procyanidins with as many as 10 flavanol subunits have been identified in cocoa and chocolate. Studies with subjects given flavanol- and procyanidin-rich foods have reported positive vascular effects including increased plasma antioxidant status (Cao et al. 1998, Serafini et al. 1998, Leenan et al. 2000, Wang et al. 2000, Wan et al. 2001), improved endothelium function (Stein et al. 1999, Duffy et al. 2001), and reduced platelet reactivity (Keevil et al. 2000, Rein et al. 2000a,b,, Freedman et al. 2001, Holt et al. 2002, Pearson et al. 2002). It is important to note that the amount of flavonoids and flavanols in cocoa and chocolate can vary widely as a result of a multitude of factors. Agronomic factors and genetics determine the flavonoid content of plants prior to harvest. After harvest, fermentation and other processes, including roasting and alkalization, can reduce the final flavonoid content of the product. Thus, depending on harvesting and processing about 10% of the flavonoids are preserved in the final cocoa and chocolate products (Kim and Keeney 1984, Porter et al. 1991, Hannum and Erdman 2000, Keen et al. 2002).

### *Platelet reactivity after cocoa consumption*

In a series of experiments, cocoa was given to healthy adult volunteers. Prior to the experiment, subjects were instructed to abstain from ASA, and other non-steroidal anti-inflammatory medications for at least 4–6 days, from alcoholic beverages for at least 2 days, and from caffeine- or theobromine-containing foods and flavonoid-rich foods for at least 24 h prior to and during the test days. On the day of the experiment, all volunteers were given flavonoid-rich cocoa that provided 897 mg of total cocoa procyanidins as determined by Adamson et al. (1999). Venous blood was drawn prior to consumption of the flavonoid-rich cocoa 2 and 6 h post consumption. Whole blood was activated, *ex vivo*, with known platelet agonists epinephrine (20 μmol/l), or adenosine diphosphate (ADP; 20 μmol/l), or no stimulation, and in the presence of fluorescent-labeled antibodies to the platelet surface proteins P-Selectin and the activated conformation of glycoprotein (GP) IIb/IIIa. The level of surface protein expression was detected using flow cytometry and the labeled platelets (Rein et al. 2000a,b, Pearson et al. 2002). Platelet-related primary hemostasis was also determined in whole blood by using a platelet function analyzer (PFA-100™) (Rein et al. 2000a, Pearson et al. 2002). In additional experiments, the effects of flavonoid-rich cocoa consumption on platelet activation and function were compared to the consumption of dealcholized red wine (DRW), a caffeine-containing beverage, and to the known platelet inhibitor aspirin (ASA) (Rein et al. 2000b, Pearson et al. 2002).

Upon activation by agonists such as collagen, thrombin, ADP or epinephrine, platelets undergo a series of intracellular signaling steps that convert the GP IIb/IIIa receptor from a resting state on the platelet surface to an active conformation that binds fibrinogen and von Willebrand factor (vWF), leading to platelet aggregation (Topol et al. 1999). Antagonists of ligand binding to the GP IIb/IIIa receptor have been effectively used in patients undergoing percutaneous coronary interventions to minimize further ischemic episodes (Topol et al. 1999, Bhatt and Topol 2003). An extract of green tea flavanols (catechin, epicatechin, epigallocatechin, gallocatechin, gallocatechin gallate and epicatechin gallate) reduced collagen-, ADP- and thrombin-induced platelet aggregation, and GP IIb/IIIa expression in ADP-stimulated platelets, *in vitro* (Kang et al. 2001). We, therefore, examined the effect of flavonoid-rich cocoa consumption on the activated conformation of GP IIb/IIIa. A decrease in epinephrine-stimulated GP IIb/IIIa expression was observed 2 (Rein et al. 2000a,b, Pearson et al. 2002) and 6 h (Rein et al. 2000a,b) after subjects consumed the cocoa. A similar effect was also observed with ADP stimulation (Rein et al. 2000a,b). In subjects given DRW, water or a caffeine containing drink, no significant changes in either epinephrine (20 μmol/l) or ADP (20 μmol/l) stimulation of GP IIb/IIIa were observed. When a higher concentration of agonist was used (100 μmol/l ADP), a significant increase in GP IIb/IIIa expression was produced after DRW consumption (Table I).

P-Selectin, which is expressed on the surface of activated platelets after degranulation, acts to mediate platelet and leukocyte adherence, and has been examined as a potential marker for assessment of CVD risk (Parker et al. 2001, Romuk et al. 2002). In subjects given cocoa, P-Selectin was reduced using either ADP (Rein et al. 2000b) or epinephrine (Rein et al. 2000a) stimulation 2 and 6 h after flavonoid-rich cocoa consumption. No effect on platelet ADP or epinephrine P-Selectin expression was observed when subjects were given DRW or water (Rein et al. 2000a,b), however, an increase in epinephrine stimulated platelet reactivity was observed when subjects were given a caffeine-rich drink. These results

Table I. The effects of DRW and cocoa procyanidins on *in vivo* platelet activation in whole blood.

| | Glycoprotein IIb/IIIa expression | | | |
|---|---|---|---|---|
| | Unstimulated | Epinephrine (2 μM) | ADP (2 μM) | ADP (100 μM) |
| Water | – | – | – | – |
| Cocoa | ↓2 and 6 h ($p = 0.035$) | ↓2 and 6 h ($p = 0.008$) | ↓2 and 6 h ($p = 0.001$) | – ($p = 0.067$) |
| DRW | – | – | – | ↑2 and 6 h ($p = 0.033$) |
| Caffeine | – | ↑2 and 6 h ($p = 0.048$) | – ($p = 0.066$) | – |

Table represents significant trends in glycoprotein IIb/IIIa expression in epinephrine-stimulated (20 μmol/l) whole blood after healthy human subjects were provided 300 ml of the test foods. Dashes represent no observed effect; otherwise *p* value is reported (Friedman's repeated-measures ANOVA on ranks and Tukey's all-pairwise multiple-comparison test) (Rein *et al.*, 2000).

have since been confirmed by Murphy et al. (2003), who also observed reduced P-Selectin expression after subjects were given 234 mg/day of extracted flavonoids from cocoa in tablet form for 28 days. In addition, Hodgson et al. (2001) reported reductions in soluble P-Selectin levels in the plasma after subjects were given 5 cups of black tea/day for 4 weeks.

Microparticles are membrane vesicles that are shed from platelets upon activation, and exhibit procoagulant activity (Horstman and Ahn 1999). A significant increase in platelet microparticles that was also correlated to P-Selectin expression was recently reported in patients with severe hypertension (Preston et al. 2003). In a healthy population of subjects, consumption of flavonoid-rich cocoa reduced the amount of microparticles formed 2 and 6 h post consumption (Rein et al. 2000a), conversely, subjects given water and water with caffeine showed increased microparticle formation (Figure 1).

The PFA-100™ measures platelet-related primary hemostasis as the time it takes for ADP or epinephrine stimulated whole blood to occlude an aperture in a collagen membrane under simulated small vessel shear conditions (Kundu et al. 1996, Fressinaud et al. 1998). In the cocoa feeding studies, an increase in the PFA-100™ closure time in seconds indicated a reduced propensity of platelets to adhere and aggregate. Cocoa consumption increased epinephrine-collagen closure times 6 h after subjects were given cocoa (Rein et al. 2000, Pearson et al. 2002). In addition, cocoa consumption increased ADP-collagen stimulated closure times 2 h post consumption (Pearson et al. 2002). The caffeine-containing beverage also prolonged ADP-collagen stimulated closure times 6 h post consumption (Rein et al. 2000). It is important to note that it has been recently reported that different platelet function assays have varying sensitivities depending on the drug regime (Hezard et al. 2003), thus it is important to assess the effects of flavonoids on platelet function using a variety of assays. While black tea consumption has shown either no (Duffy et al. 2001, Hodgson et al. 2001) or a positive effect (Wolfram et al. 2002) on platelet function using platelet rich plasma (PRP) and aggregometry, cocoa flavonoid consumption has been shown to reduce platelet function using both whole-blood aggregometry (Murphy et al. 2003) and the PFA-100™ (Rein et al. 2000a, Holt et al. 2002, Pearson et al. 2002). Likewise, grape products, such as grape juice, grape skin and seed extracts, and red wine, have all been shown to reduce platelet reactivity by using whole blood aggregometry (Osman et al. 1998, Keevil et al. 2000, Freedman et al. 2001, Shanmuganayagam et al. 2002) or by cyclic flow reduction (Demrow et al. 1995).

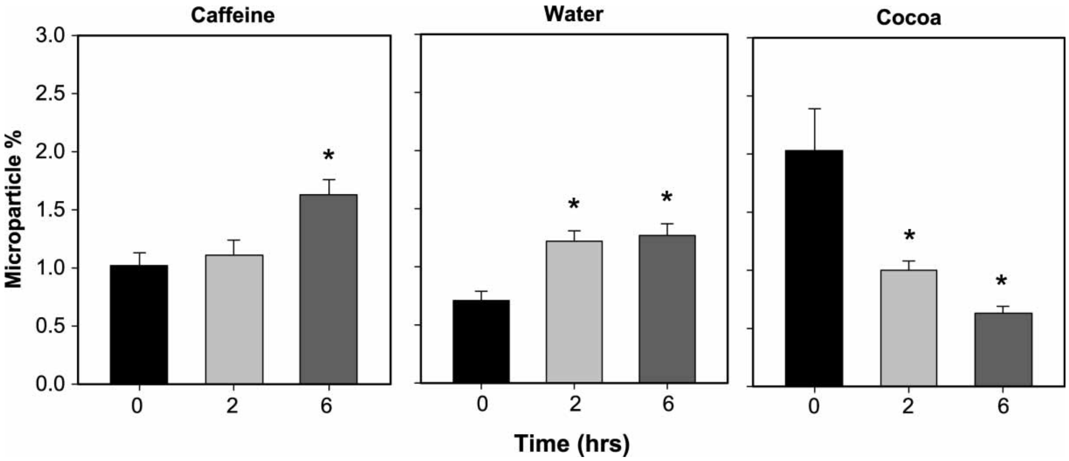

Figure 1. Platelet microparticle formation in response to the consumption of cocoa, water, or a caffeine beverage. Blood was drawn before (0 h) and 2 and 6 h after the consumption of the indicated beverages. Data are presented as the percentage of platelet microparticles of total CD42-positive events (mean ± SEM). *Significantly different from baseline (0 h) with each treatment (Friedman's repeated-measures ANOVA on ranks and Tukey's all-pairwise multiple-comparison test) (Rein et al. 2000a).

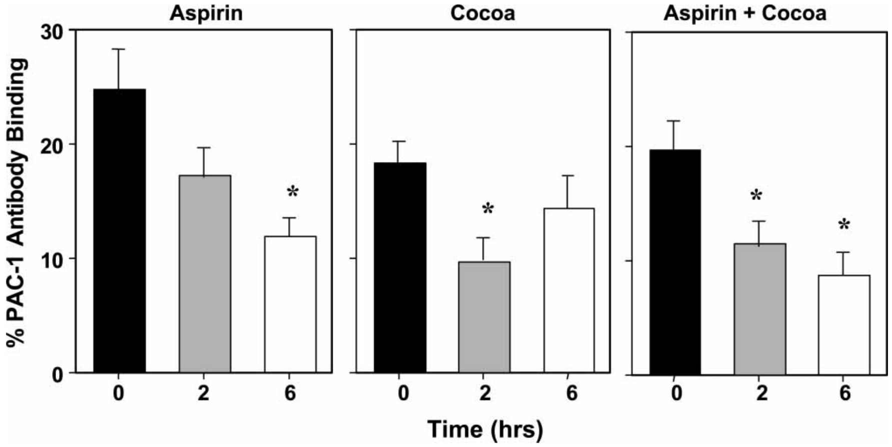

Figure 2. Platelet activation in response to the consumption of cocoa, aspirin (ASA; 81 mg) or ASA + cocoa. Blood was drawn before (0 h) and 2 and 6 h after the consumption of the test compounds. The percentage of platelets expressing activated GP IIb/IIIa (% PAC-1 antibody binding) in whole blood after stimulation with 20 μmol/l epinephrine; $n = 16$ in each group. Data are presented as mean ± SEM. *Significantly different from baseline (0 h) with each treatment, $p \leq 0.05$ (Friedman's repeated-measures ANOVA on ranks and Tukey's all-pairwise multiple-comparison test) (Pearson et al. 2002).

Aspirin (ASA) permanently inactivates cyclo-oxygenase-1 (COX-1) through selective acetylation of one of its serine residues (Patrono 1994). Platelets lack the ability to regenerate this enzyme, thus resulting in the irreversible loss of the potent aggregator, thromboxane, for the lifespan of the platelet (8–10 days). When the effects of cocoa were compared to ASA use (Pearson et al. 2002), GP IIb/IIIa expression on ADP-activated platelets was reduced 6 h after cocoa and ASA were taken together, but not when either cocoa or ASA were given to subjects separately. ASA was able to reduce the surface expression of GPIIb/IIIa on epinephrine-activated platelets 6 h post consumption, while cocoa reduced expression 2 h post consumption. However, when given together, ASA and cocoa consumption was able to decrease the expression of GP IIb/IIIa both 2 and 6 h post consumption after epinephrine stimulation (Figure 2). Similar results were observed with P-Selectin expression. When given separately, only ASA was able to reduce P-Selectin expression (6 h), however, when given together, both cocoa and ASA were able to reduce P-selectin expression 2 and 6 h after administration (Pearson et al. 2002).

With regards to platelet-related primary hemostasis, cocoa increased PFA closure-times 2 h post consumption. As predicted, ASA had no effect on ADP-collagen stimulation, however, when taken together both cocoa and ASA significantly increased closure times 2 and 6 h after administration. ASA significantly increased epinephrine-collagen PFA closure time 2 and 6 h post consumption, while cocoa only increased closure time 2 h post consumption. Similar to ADP-collagen stimulation, when ASA and cocoa were given together, epinephrine-collagen PFA closure times were significantly increased from baseline both 2 and 6 h post consumption (Figure 3) (Pearson et al. 2002).

In the above study, a rather large procyanidin (800 mg) dose was given to the subjects. In order to determine the amount of procyanidin necessary to produce the observed platelet effects subjects were given a smaller flavanol load (200 mg) in the form of 25 g of semi-sweet chocolate chips. ADP-collagen and epinephrine-collagen stimulation prolonged closure times 2 h post-consumption, while 6 h post consumption, only ADP-collagen stimulation prolonged closure times (Holt et al. 2002).

### Potential mechanism regarding the effects of flavonoids on platelet reactivity

The ability of flavanol-rich cocoa to favorably modulate platelet reactivity was consistently demonstrated. By several measures, ADP- and epinephrine-induced GP IIb/IIIa and P-Selectin expression, ADP- and epinephrine-collagen induced primary hemostasis, and microparticle formation, flavanol-rich cocoa inhibited platelet reactivity. For the most part, these effects were not seen with a caffeine-containing beverage, suggesting that the caffeine component of cocoa was not responsible for the observed effects of the cocoa beverage. Furthermore, the inhibitory effects of flavanol-rich cocoa on epinephrine-induced GP IIb/IIIa expression and primary hemostasis were less profound, but similar to those seen with low dose ASA.

Recently, intraperitoneal injections of catechin were reported to inhibit radical-induced platelet hyperactivity (Blaiche et al. 2002). Although cocoa contains catechin, it is still unclear the specific or combination of cocoa components that are responsible for the observed *ex vivo* platelet-inhibitory effects. Another recent study observed a greater platelet inhibitory effect when grape seed and grape skin extracts were

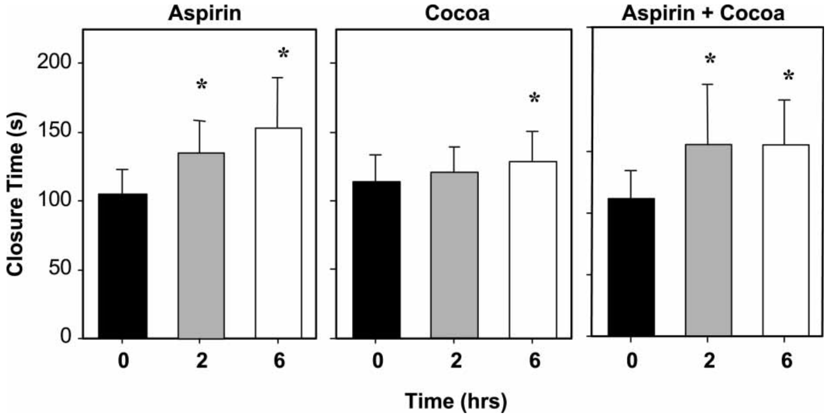

Figure 3. Platelet-dependent primary hemostasis in response to the consumption of cocoa, aspirin (ASA; 81 mg) or ASA + cocoa as measured by a platelet function analyzer (PFA-100™). Blood was drawn before (0 h) and 2 and 6 h after the consumption of the test compounds. Whole blood was aspirated through an aperture coated with collagen-epinephrine and the time required to occlude the aperture measured as the closure time in seconds. Data are presented as mean ± SD. *Significantly different from baseline (0 h) within each treatment, $p \leq 0.0005$, (see text for individual $p$ values), (Friedman's repeated-measures ANOVA on ranks and Tukey's all-pairwise multiple-comparison test) (Pearson et al. 2002).

given at the same time compared to when provided separately (Shanmuganayagam et al. 2002). The cocoa procyanidins, trimer and pentamer (3 μmol/l), have been shown to inhibit epinephrine-stimulated GP IIb/IIIa, *in vitro* (Rein et al. 2000), and is consistent with the observation that cocoa consumption inhibits epinephrine-induced GP IIb/IIIa expression. Although these data suggest that the cocoa trimer and pentamer procyanidins are possible platelet-inhibitory components, their comparison to *in vivo* observations must be tempered by the fact that only flavanols and dimeric procyandins have been detected in the circulation and the urine (Piskula and Terao 1998, Donovan et al. 1999, Richelle et al. 1999, Baba et al. 2000, Holt et al. 2002), and mostly as conjugated metabolites. In addition, although procyanidins may survive the acidic conditions of the stomach (Rios et al. 2002) recent data suggests that they may be absorbed as lower molecular weight phenolic acids after microbial degradation (Rios et al. 2003). Thus, further research is needed to fully characterize flavanols, and their metabolites, for their platelet-inhibitory effects.

Adding complexity to platelet-flavonoid research is the fact that several agonists trigger platelet activation, adhesion and aggregation, all with multiple signaling pathways that modulate or amplify each of these steps. Thus, the possibility exists, and research suggests, that specific flavonoids and flavonoid-rich foods can affect platelet function at different steps and via different mechanisms. In the above studies, DRW exhibited no platelet inhibitory effect and actually was stimulatory at a higher ADP (100 μmol/l) concentration (Rein et al. 2000), yet other studies have demonstrated an inhibitory effect of red wine on platelet function. For example, studies have shown that red wine consumption can inhibit *ex vivo* ADP- (Seigneur et al. 1990) and thrombin-induced (Pace-Asciak et al. 1996) platelet aggregation in platelet-rich plasma. Similarly, red wine and grape juice have been found to suppress cyclic flow reductions in stenosed canine arteries (Demrow et al. 1995, Osman et al. 1998), as well as, inhibit *ex vivo* collagen-induced platelet aggregation following their consumption by human volunteers (Keevil et al. 2000, Freedman et al. 2001). This platelet-inhibitory effect seen with grape juice was not observed after orange or grapefruit juice consumption (Osman et al. 1998, Keevil et al. 2000). Like red wine and cocoa, grape juice contains significant amounts of flavonoids, particularly the flavanols and flavonols, whereas orange and grapefruit juice, while containing other classes of polyphenolic compounds, have minimal amounts of flavanols.

Not all studies have demonstrated platelet-inhibitory effects following the consumption of flavonoid-rich foods and beverages. Duffy et al. reported that both acute and chronic consumption of black tea had no effect on platelet aggregation (Duffy et al. 2001). The lack of effect of black tea reported by Duffy et al. may be in part due to the subject's daily dose of 325 mg ASA, a large enough dose that may have masked any subtle, but still important effects of the black tea. It is important to note that, ADP and thrombin receptor-activating peptide were used as agonists, thus, a lack of effect of black tea in this study does not preclude an effect with other biologically relevant agonists. In addition, while grapes and cocoa contain flavanols and procyanidins, tea contains flavanols, flavanol galloyl esters, theaflavins and thearubigins (Kris-Etherton and Keen 2002). Thus, future studies are needed to elucidate the effects of specific flavanols, and different classes of flavanol oligomers on platelet function.

The exact mechanism(s) by which flavanols inhibit platelet activity was not addressed in our series of studies, although several possibilities exist. The well-known antioxidant activities of numerous flavonoids (Rice-Evans et al. 1996), including the flavanols found in cocoa (Sanbongi et al. 1998, Harada et al. 1999, Zhu et al. 2002), may mediate their platelet effects. Catechin alone, and synergistically with quercetin, has been shown to decrease collagen-induced hydrogen peroxide production, and inhibit collagen-induced platelet aggregation and adhesion (Pignatelli et al. 2000). As mentioned earlier, catechin has been observed to inhibit iron-induced platelet hyperactivity in rats, including a decrease in $F_2$-isoprostanes, TBARS formation, and vitamin E sparing, all suggestive of an antioxidant effect of catechin (Blaiche et al. 2002). Superoxide anion is known to enhance platelet aggregation, and can react with nitric oxide to form reactive nitrogen species, thus, decreasing the antithrombotic activity of nitric oxide (Handin et al. 1977, Ohara et al. 1993). Freedman et al. reported that purple grape juice consumption increased plasma protein-independent antioxidant activity, coupled with a decrease in superoxide production, thus, suggesting that the mechanism behind reduced platelet aggregation is through an increase in platelet-derived nitric oxide production (Freedman et al. 2001).

Although these studies suggest that flavanols may exert their platelet effects through an antioxidant mechanism, they may also act through additional non-antioxidant mechanisms, such as cell signaling pathways and eicosanoid metabolism. For example, purple grape juice has been reported to attenuate platelet protein kinase C activity (Freedman et al. 2001), while catechin and quercetin have been observed to reduce phospholipase C activity (Pignatelli et al. 2000). Several studies have reported the ability of individual flavonoids, or flavonoid-rich foods, to modulate eicosanoid metabolism, including an increase ratio of plasma prostacyclin to leukotriene (Schramm et al. 2001, Holt et al. 2002), and reduced thromboxane production (Corvazier and Maclouf 1985,Chang and Hsu 1989, Blaiche et al. 2002). In addition, flavanols and procyanidins from cocoa were shown to inhibit platelet 12-lipoxygenase (Schewe et al. 2001) and 5-lipoxygenase (Schewe et al. 2002). Collectively, these data suggest that flavanols may mediate platelet function through a multitude of mechanisms.

In summary, research indicates that the flavanols found in cocoa, as well as other flavanol-rich foods, favorably modulate platelet function. In light of the fact that CVD remains as the number one cause of mortality in the US, coupled with a decline in flavonoid-rich foods (i.e. fruits and vegetables) consumption over the past few decades (Patterson et al. 1990, Block 1991), it becomes increasingly warranted to study a variety of flavanoid-rich foods for possible positive vascular effects. In particular, continued research is needed to characterize the mechanisms of platelet action of the biologically present forms of flavanols. Clinical trials are needed that use a variety of platelet function assays that are clinically correlated with thromboembolic risk, to fully determine their role in cardiovascular disease prevention and intervention. With a more complete understanding of the physiologic effects of flavanol-rich foods, future dietary guidelines can be refined and or modified to optimize the health benefits of these compounds.

### References

Adamson GE, Lazarus SA, Mitchell AE, et al. 1999. HPLC method for the quantification of procyanidins in cocoa and chocolate samples and correlation to total antioxidant capacity. J Agric Food Chem 47:4184–4188.

Apitz-Castro R, Badimon JJ, Badimon L. 1992. Effect of ajoene, the major antiplatelet compound from garlic, on platelet thrombus formation. Thromb Res 68:145–155.

Baba S, Osakabe N, Yasuda A, et al. 2000. Bioavailability of (−)-epicatechin upon intake of chocolate and cocoa in human volunteers. Free Radic Res 33:635–641.

Bhatt D, Topol E. 2000. Current role of platelet glycoprotein IIb/IIIa inhibitors in acute coronary syndromes. J Am Med Assoc 284:1549–1558.

Bhatt DL, Topol EJ. 2003. Scientific and therapeutic advances in antiplatelet therapy. Nat Rev: Drug Disc 2:15–28.

Blaiche D, Durand P, Prost M, Loreau N. 2002. (+)-Catechin inhibits platelet hyperactivity induced by an acute iron load *in vivo*. Free Radic Biol Med 33:1670–1680.

Blair IA, Dougherty RM, Iacono JM. 1994. Dietary stearic acid and thromboxane-prostacyclin biosynthesis in normal human subjects. Am J Clin Nutr 60:1054S–1058S.

Block G. 1991. Dietary guidelines and the results of food consumption surveys. Am J Clin Nutr 53:356S–357S.

Cao G, Russell R, Lischner N, Prior R. 1998. Serum antioxidant capacity is increased by consumption of strawberries, spinach, red wine or vitamin C in elderly women. J Nutr 128:2383–2390.

Chang W-C, Hsu F-L. 1989. Inhibition of platelet aggregation and arachidonate metabolism in platelets by procyanidins. Prostaglandins Leukot Essent Fatty Acids 38:181–188.

Chevaux KA, Jackson L, Villar ME, et al. 2001. Proximate, mineral and procyanidin content of certain foods and beverages consumed by the Kuna Amerinds of Panama. J Food Compos Anal 14:553–563.

Corvazier E, Maclouf J. 1985. Interference of some flavonoids and non-steroidal anti-inflammatory drugs with oxidative metabolism of arachidonic acid by human platelets and neutrophils. Biochem Biophys Acta 835:315–321.

Demrow HS, Slane PR, Folts JD. 1995. Administration of wine and grape juice inhibits *in vivo* platelet activity and thrombosis in stenosed canine coronary arteries. Circulation 91:1182–1188.

Demrow H, Slane P, Folts J. 1995. Administration of wine and grape juice inhibits *in vivo* platelet activity and thrombosis in stenosed canine coronary arteries. Circulation 15:1182–1188.

Donovan JL, Bell JR, Kasim-Karakas S, et al. 1999. Catechin is present as metabolites in human plasma after consumption of red wine. J Nutr 129:1662–1668.

Duffy S, Vita J, Holbrook M, Swerdloff P, Keaney JF, Jr. 2001. Effect of acute and chronic tea consumption on platelet aggregation in patients with coronary artery disease. Arterioscler Thromb Vasc Biol 21:1084–1089.

Freedman JE, Parker C, III, Li L, et al. 2001. Select flavonoids and whole juice from purple grapes inhibit platelet function and enhance nitric oxide release. Circulation 103:2792–2798.

Fressinaud E, Veyradier A, Truchaud F, et al. 1998. Screening for von Willebrand disease with a new analyzer using high shear stress: a study of 60 cases. Blood 91:1325–1331.

Hammerstone JF, Lazarus SA, Mitchell AE, Rucker R, Schmitz HH. 1999. Identification of procyanidins in cocoa (*Theobroma cacao*) and chocolate using high-performance liquid chromatography/mass spectrometry. J Agric Food Chem 47:490–496.

Handin R, Karabin R, Boxer G. 1977. Enhancement of platelet function by superoxide anion production. J Clin Investig 59:959–965.

Hannum SM, Erdman JJW. 2000. Emerging health benefits from cocoa and chocolate. J Med Food 3:73–75.

Harada M, Kan Y, Naoki H, et al. 1999. Identification of the major antioxidative metabolites in biological fluids of the rat with ingested (+)-catechin and (−)-epicatechin. Biosci Biotechnol Biochem 63:973–977.

Hennekens CH. 1997. Aspirin in the treatment and prevention of cardiovascular disease. Annu Rev Public Health 18:37–49.

Hezard N, Metz D, Nazeyrollas P, Droulle C, Potron G, Nguyen P. 2003. PFA-100™ and flow cytometry: Can they challenge aggregometry to assess antiplatelet agents, other than GPIIbIIIa blockers, in coronary angioplasty? Thromb Res 108:43–47.

Hodgson J, Puddey IB, Mori T, Burke V, Baker R, Beilin L. 2001. Effects of regular ingestion of black tea on haemostasis and cell adhesion molecules in humans. Eur J Clin Nutr 55:881–886.

Holt RR, Schramm DD, Keen CL, Lazarus SA, Schmitz H. 2002. Flavonoid-rich chocolate and platelet function. J Am Med Assoc 287:2212–2213.

Holt RR, Lazarus SA, Sullards M, et al. 2002. Procyanidin dimer B2 (epicatechin-(4b-8)-epicatechin) in human plasma after the consumption of a flavanol-rich cocoa. Am J Clin Nutr 76:798–804.

Hook JC. 1994. Stearic acid, clotting and thrombosis. Am J Clin Nutr 60:1050S–1053S.

Horstman LL, Ahn YS. 1999. Platelet microparticle: a wide angle perspective. Crit Rev Oncol/Hematol 30:111–141.

Hu FB, Willett WC. 2002. Optimal diets for prevention of coronary heart disease. J Am Med Assoc 288:2569–2578.

Hughes A, Tonks R. 1966. Magnesium, adenosine diphosphate and blood platelets. Nature 210:106–107.

Kang W-S, Chung K-H, Chung J-H, et al. 2001. Antiplatelet activity of green tea catechins is mediated by inhibition of cytoplasmic calcium increase. J Cardiovasc Pharmacol 38:875–884.

Keen CL, Holt RR, Polagruto JA, Wang JF, Schmitz HH. 2002. Cocoa flavanols and cardiovascular health. Phytochem Rev 1:231–240.

Keevil JG, Osman HE, Reed JD, Folts JD. 2000. Grape juice, but not orange juice or grapefruit juice, inhibits human platelet aggregation. J Nutr 130:53–56.

Kim H, Keeney P. 1984. (−)-Epicatechin content in fermented and unfermented cocoa beans. J Food Sci 49:1090–1092.

Krauss RM, Eckel RH, Howard B, et al. 2000. AHA dietary guidelines Revision 2000: A statement for healthcare professionals from the nutrition committee of the American Heart Association. Circulation 102:2284–2299.

Kris-Etherton P, Keen CL. 2002. Evidence that the antioxidant flavonoids in tea and cocoa are beneficial for cardiovascular health. Curr Opin Lipidol 13:41–49.

Kris-Etherton P, Mustad VA. 1994. Chocolate feeding studies: A novel approach for evaluating the plasma lipid effects of stearic acid. Am J Clin Nutr 60:1029S–1036S.

Kundu S, Heilmann E, Sio R, Garcia C, Ostagaard R. 1996. Characterization of an *in vitro* platelet function analyzer, PFA-100. Clin Appl Thromb Hemostasis 2:241.

Leenan R, Roodenburg A, Tijburg L, Wiseman S. 2000. A single dose of tea with or without milk increases plasma antioxidant activity in humans. Eur J Clin Nutr 54:87–92.

Lin H, Young D. 1994. Interaction between plasma potassium and epinephrine in coronary thrombosis in dogs. Circulation 89:331–338.

Ma G, Young DB, Clower BR, Anderson PG, Lin H, Abide AM. 2000. High potassium intake inhibits neointima formation in the rat carotid artery balloon injury model. Am J Hypertens 13:1014–1020.

Ma G, Srivastava N, Anderson P, et al. 2001. Elevated potassium intake inhibits neointimal proliferation in the swine coronary artery. Am J Hypertens 14:879–886.

Mensink RP, Zock PL, Kester AD, Katan MB. 2003. Effects of dietary fatty acids and carbohydrates on the ratio of serum total to HDL cholesterol and on serum lipids and apolipoproteins: A meta-analysis of 60 controlled trials. Am J Clin Nutr 77:1146–1155.

Middleton E, Jr., Kandaswami C, Theoharides TC. 2000. The effects of plant flavonoids on mammalian cells: Implications for inflammation, heart disease, and cancer. Pharmacol Rev 52:673–751.

Mitropoulos K, Miller G, Martin J, Reaves B, Cooper J. 1994. Dietary fat induces changes in factor VII coagulant activity through effects on plasma free stearic acid concentration. Arterioscler Thromb 14:214–222.

Murphy KJ, Chronopoulos AK, Singh I, et al. 2003. Dietary flavanols and procyanidin oligomers from cocoa (*Theobroma cacao*) inhibit platelet function. Am J Clin Nutr 77:1466–1473.

Mustad VA, Kris-Etherton P, Derr J, Reddy C, Pearson TA. 1993. Comparison of the effects of diets rich in stearic acid versus myristic acid and lauric acid on platelet fatty acids and excretion of thromboxane A2 and PGI2 metabolites in healthy young men. Metabolism 42:463–469.

Ohara Y, Peterson T, Harrison D. 1993. Hypercholesterolemia increases endothelial superoxide anion production. J Clin. Investig 91:2546–2551.

Osman HE, Maalej N, Shanmuganayagam D, Folts J. 1998. Grape juice but not orange or grapefruit juice inhibits platelet activity in dogs and monkeys. J Nutr 128:2307–2312.

Osterud B. 1997. A global view on the role of monocytes and platelets in atherogenesis. Thromb. Res 85:1–22.

Otsuka K, Watanabe M, Yue Q, McCarron DA, Hatton D. 1997. Dietary calcium attenuates platelet aggregation and intracellular $Ca^{2+}$ mobilization in spontaneously hypertensive rats. Am J Hypertens 10:1165–1170.

Otton R, Graziola F, De Souza JA, Curi TC, Hirata MH, Curi R. 1998. Effect of dietary fat on lymphocyte proliferation and metabolism. Cell Biochem Funct 16:253–259.

Pace-Asciak C, Rounova O, Hahn S, Diamandis E, Goldberg D. 1996. Wines and grape juices as modulators of platelet aggregation in healthy human subjects. Clin Chim Acta 15:163–182.

Parker C, III, Vita J, Freedman JE. 2001. Soluble adhesion molecules and unstable coronary artery disease. Atherosclerosis 156:417–424.

Patrono C. 1994. Aspirin as an antiplatelet drug. N. Engl. J Med 330:1287–1294.

Patterson B, Block G, Rosenberger W, Pee D, Kahle L. 1990. Fruit and vegetables in the American diet: data from the NHANES II survey. Am J Public Health 80:1443–1449.

Pearson D, Paglieroni T, Rein D, et al. 2002. The effects of flavanol-rich cocoa and aspirin on *ex vivo* platelet function. Thromb Res 106:191–197.

Petrov V, Lijnen P. 1999. Modification of intracellular calcium and plasma renin by dietary calcium in men. Am J Hypertens 12:1217–1224.

Pignatelli P, Pulcinelli FM, Celestini A, et al. 2000. The flavonoids quercetin and catechin synergistically inhibit platelet function by antagonizing the intracellular production of hydrogen peroxide. Am J Clin Nutr 72:1150–1155.

Piskula MK, Terao J. 1998. Accumulation of (−)-epicatechin metabolites in rat plasma after oral administration and distribution of conjugation enzymes in rat tissues. J Nutr 128:1172–1178.

Planells E, Rivero M, Carbonell J, Mataix J, Llopis J. 1997. Ability of a cocoa product to prevent chronic Mg deficiency in rats. J Agric Food Chem 45:4017–4022.

Porter LJ, Ma Z, Chan B. 1991. Cacao procyanidins: Major flavanoids and identification of some minor metabolites. Phytochemistry 30:1657–1663.

Preston RA, Jy W, Jimenex JJ, et al. 2003. Effects of severe hypertension on endothelial and platelet microparticles. Hypertension 41:211–217.

Rauch U, Osende JI, Fuster V, Badimon JJ, Fayad Z, Chesebro JH. 2001. Thrombus formation on atherosclerotic plaques: Pathogenesis and clinical consequences. Ann Intern Med 134:224–238.

Rein D, Paglieroni TG, Wun T, et al. 2000a. Cocoa inhibits platelet activation and function. Am J Clin Nutr 72:30–35.

Rein D, Paglieroni TG, Pearson DA, et al. 2000b. Cocoa and wine polyphenols modulate platelet activation and function. J Nutr 130:2120S–2126S.

Rice-Evans CA, Miller NJ, Paganga G. 1996. Structure-antioxidant activity relationships of flavonoids and phenolic acids. Free Radic Biol Med 20:933–956.

Richelle M, Tavazzi I, Enslen M, Offord E. 1999. Plasma kinetics in man of epicatechin from black chocolate. Eur J Clin Nutr 53:22–26.

Rios L, Bennett R, Lazarus S, Remesy C, Scalbert A, Williamson G. 2002. Cocoa procyanidins are stable during gastric transit in humans. Am J Clin Nutr 76:1106–1110.

Rios LY, Gonthier M-P, Remesy C, et al. 2003. Chocolate intake increases urinary excretion of polyphenol-derived phenolic acids in healthy human subjects. Am J Clin Nutr 77:912–918.

Romuk E, Skrzep-Poloczek B, Wojciechowska C, et al. 2002. Selectin-P and interleukin-8 plasma levels in coronary heart disease patients. Eur J Clin Investig 32:657–661.

Ruggeri ZM. 2002. Platelets in atherothrombosis. Nat. Med 8:1227–1234.

Saamman S, Lai NT, Sullivan DR. 2000. The effect of a lipid-lowering diet on plasma lipids and lipoproteins in mildly hypercholesterolaemic subjects: A potential role for occasional treats. J Nutr Biochem 11:250–254.

Sanbongi C, Osakabe N, Nausume M, Takizawa T, Gomi S, Osawa T. 1998. Antioxidative polyphenols isolated from theobroma cacao. J Agric Food Chem 46:454–457.

Schafer A. 1996. Antiplatelet therapy. Am J Med 101:199–209.

Schewe T, Sadik C, Klotz L-O, Yoshimoto T, Kuhn H, Sies H. 2001. Polyphenols of cocoa: Inhibition of mammalian 15-lipoxygenase. Biol Chem 382:1687–1696.

Schewe T, Kuhn H, Sies H. 2002. Flavonoids of cocoa inhibit recombinant human 5-lipoxygenase. J Nutr 132:1825–1829.

Schramm DD, Wang JF, Holt RR, et al. 2001. Chocolate procyanidins decrease the leukotriene-prostacyclin ratio in humans and human aortic endothelial cells. Am J Clin Nutr 73:36–40.

Seigneur M, Bonnet J, Dorian B. 1990. Effect of the consumption of alcohol, white wine, and red wine on platelet function and serum lipids. J Appl. Cardiol 5:215–222.

Serafini M, Maiani G, Ferro-Luzzi A. 1998. Alcohol-free red wine enhances plasma antioxidant capacity in humans. J Nutr 128:1003–1007.

Shanmuganayagam D, Beahm MR, Osman HE, Krueger CG, Reed JD, Folts JD. 2002. Grape seed and grape skin extracts elicit a greater antiplatelet effect when used in combination than when used individually in dogs and humans. J Nutr 132:3592–3598.

Sheu J-R, Hsiao G, Shen M-Y, et al. 2002. Mechanisms involved in the antiplatelet activity of magnesium in human platelets. Br. J Haematol 119:1033–1041.

Srivastava K. 1986. Onion exerts antiaggregatory effects by altering arachindonic acid metabolism in platelets. Prostaglandins Leukot. Med 24:43–50.

Stein JH, Keevil JG, Wiebe DA, Aeschlimann S, Folts J. 1999. Purple grape juice improves endothelial function and reduces the susceptibility of LDL cholesterol to oxidation in patients with coronary artery disease. Circulation 100:1050–1055.

Steinberg FM, Bearden MM, Keen CL. 2003. Cocoa and chocolate flavonoids: implications for cardiovascular health. J Am Diet Assoc 103:215–223.

Tholstrup T, Marckmann P, Jespersen J, Sandstrom B. 1994. Fat high in stearic acid favorably affects blood lipids and factor VII coagulant activity in comparison with fats high in palmitic acid or high in myristic and lauric acids. Am J Clin Nutr 59:371–377.

Topol EJ, Byzova TV, Plow EF. 1999. Platelet GPIIB-IIIa blockers. Lancet 353:227–231.

Wan Y, Vinson JA, Etherton TD, Proch J, Lazarus S, Kris-Etherton P. 2001. Effects of cocoa powder and dark chocolate on LDL oxidative susceptibility and prostaglandin concentrations in humans. Am J Clin Nutr 74:596–602.

Wang JF, Schramm DD, Holt RR, et al. 2000. A dose–response effect from chocolate consumption on plasma epicatechin and oxidative damage. J Nutr 130:2115S–2119S.

Whelton PK, Jiang H, Appel LJ, et al. 2002. Primary prevention of hypertension. Clinical and public health advisory from the National High Blood Pressure Education Program. J Am Med Assoc 288:1882–1888.

Wolfram R, Oguogho A, Efthimiou Y, Budinsky A, Sinzinger H. 2002. Effect of black tea on (iso-)prostaglandins and platelet aggregation in healthy volunteers. Prostaglandins Leukot Essent Fatty Acids 66:529–533.

Young D, Lin H, McCabe R. 1995. Potassium's cardiovascular protective mechanisms. Am J Physiol 268:R825–R837.

Zhu Q, Holt R, Lazarus S, Orozco T, Keen C. 2002. Inhibitory effects of cocoa flavanols and procyanidin oligomers on free radical-induced erythrocyte hemolysis. Exp Biol Med 227:321–329.

